# Use of Machine-Learning and Load–Velocity Profiling to Estimate 1-Repetition Maximums for Two Variations of the Bench-Press Exercise

**DOI:** 10.3390/sports9030039

**Published:** 2021-03-16

**Authors:** Carlos Balsalobre-Fernández, Kristof Kipp

**Affiliations:** 1 Applied Biomechanics and Sports Technology Research Group, Autonomous University of Madrid, 28049 Madrid, Spain; 2 Department of Physical Therapy—Program in Exercise Science, Marquette University, Milwaukee, WI 53233, USA; kristof.kipp@marquette.edu

**Keywords:** sports, resistance training, monitoring, regularization, neural network

## Abstract

The purpose of the current study was to compare the ability of five different methods to estimate eccentric–concentric and concentric-only bench-press 1RM from load–velocity profile data. Smith machine bench-press tests were performed in an eccentric–concentric (*n* = 192) and a concentric-only manner (*n* = 176) while mean concentric velocity was registered using a linear position transducer. Load–velocity profiles were derived from incremental submaximal load (40–80% 1RM) tests. Five different methods were used to calculate 1RM using the slope, intercept, and velocity at 1RM (minimum velocity threshold—MVT) from the load–velocity profiles: calculation with individual MVT, calculation with group average MVT, multilinear regression without MVT, regularized regression without MVT, and an artificial neural network without MVT. Mean average errors for all methods ranged from 2.7 to 3.3 kg. Calculations with individual or group MVT resulted in significant overprediction of eccentric–concentric 1RM (individual MVT: difference = 0.76 kg, *p* = 0.020, *d* = 0.17; group MVT: difference = 0.72 kg, *p* = 0.023, *d* = 0.17). The multilinear and regularized regression both resulted in the lowest errors and highest correlations. The results demonstrated that bench-press 1RM can be accurately estimated from load–velocity data derived from submaximal loads and without MVT. In addition, results showed that multilinear regression can be used to estimate bench-press 1RM. Collectively, the findings and resulting equations should be helpful for strength and conditioning coaches as they would help estimating 1RM without MVT data.

## 1. Introduction

Resistance training is paramount to increase both sports performance and overall health [1,2]. During the last decade, the association between mean concentric velocity (MCV) and the load at which that velocity is produced has been extensively analyzed as a noninvasive alternative to monitor resistance training adaptations and to prescribe resistance training loads [3,4,5,6]. One of the main reasons why load–velocity profiling has become so popular in the strength and conditioning community is because it allows for the estimation of an athlete’s 1-repetition maximum (1RM) without the need for an actual maximal test [3,7]. The association between load and MCV is very strong (i.e., coefficients of determination > 0.94), and holds across different populations (from amateur to highly trained athletes) and exercises (e.g., bench-press, squat or pull-up) [4,5]. Moreover, the strong association between load and MCV, and the relevant regression equations, hypothetically also allows researchers to estimate an athlete’s 1RM if researchers know the individual’s MCV with which they lift 1RM loads. 

The MCV at 1RM loads represents the minimum velocity threshold (MVT). It should be noted that obtaining the MVT is a major drawback of 1RM estimation via load–velocity profiling because it still necessitates a maximal test. Furthermore, MVT can vary widely among individuals [8,9] and may even change after a period of resistance training [10]. For example, using subject-specific MVT, rather than general or group-based MVT, in load–velocity profiling and 1RM estimation improve the goodness of fit and reduce the standard error of the estimate [11,12,13,14,15]. Recent research has also observed that MVT is unreliable and can significantly vary intraparticipant from one session to another [9,16]. Given that MVT is a requirement if traditional load–velocity profiles are used to estimate 1RM, this could be a potential source of error and negatively affect the appropriate prescription of resistance training loads. 

One way to overcome the limitation imposed by the requirement for subject specific MVT may be to use machine learning methods. For example, one machine learning method uses regularization (or shrinkage) of regression coefficients, which desensitizes a model’s response, improves prediction accuracy, and keeps the model from overpredicting. One common method to implement regularization is through the least absolute shrinkage and selection operator (LASSO) regression [17] (Tibshirani, 1996). From a practical perspective the use of LASSO regression for predicting 1RM from limited load–velocity data may be beneficial because the regularization of regression coefficients would keep the model from overpredicting an athletes 1RM, which may lead to overtraining through continued application of heavier than anticipated loads. Another machine learning method that may be useful for estimating 1RM from limited load–velocity data is an artificial neural network (ANN). An ANN can “learn” to model the associations between variables without explicit knowledge about their underlying relationship and can generalize to new data, even if input data are incomplete or imprecise [18] (Maier et al., 2000 Sports Engineering). With respect to estimating 1RM, ANN may be useful because they could learn to model the association between load–velocity parameters and 1RM, even if MVT are not included. The purpose of the current study is to compare the ability of five different statistical models to estimate 1RM performance of two different bench press variations from load–velocity profiling data. The five respective models were 1RM prediction with group average MVT data, individual MVT data, multilinear regression, LASSO regression, and ANN. The three latter models did not include MVT data as part of the 1RM prediction. The hypothesis was that MVT would not be needed to accurately estimate an athlete’s 1RM from load–velocity profile data.

## 2. Methods

### 2.1. Participants

Data for the current study were directly obtained from the authors of previous investigations that consisted of 116 physically active males [14,19]. Specific descriptive data for the participants are provided in Table 1. All participants had at least one year of resistance training experience with the bench-press exercise and did not report any injuries in the 6 months prior to the testing session. The study protocol complied with the Declaration of Helsinki for Human Experimentation and was approved by the Institutional Review Board at Granada University. Written informed consent was obtained from each participant before the beginning of data collection.

### 2.2. Study Procedures

The data set for the current study included a total of 368 incremental and 1RM bench-press tests [14,19]. In total, 192 of the incremental bench-press tests were performed in an eccentric–concentric manner, while the remaining 176 tests were performed in a concentric-only manner (i.e., with a 2 s pause after the barbell touched the chest). In the study by García-Ramos et al. (2020), each participant performed two 1RM bench-press press tests (one in a concentric-only manner, and another one in an eccentric–concentric manner), while in the study by Pestaña-Melero et al. (2018) each participant performed two 1RM bench-press tests in a concentric-only manner and two more in an eccentric–concentric manner for reliability purposes. All tests were performed in a Smith machine. The procedures for the incremental and 1RM bench-press tests were carried out according to current recommendations, the details of which can be found in the original articles. Briefly, however, each participant performed a standardized warm-up and an incremental protocol that consisted of performing several repetitions with loads that ranged from 40% to 100% 1RM. Then, load–velocity profiles included in the present investigation were creating using submaximal loads ranging 40–80% 1RM. Participants were instructed to perform 1–2 repetitions with their maximal intended velocity against each load. Participants were allowed 2–3 min of passive rest between sets of different loads. The mean velocity of the concentric phase of each lift (MCV) during the execution of each repetition was registered with a validated linear velocity transducer (T-Force System; Ergotech, Murcia, Spain) [20]. The MCV of the fastest repetition at each load were extracted for analysis and used for the calculation of load–velocity profiles and estimation of 1RM. In addition, the velocity at which each participant performed the actual 1RM test was also extracted—this velocity represented the minimum velocity threshold (MVT) for each participant [6].

### 2.3. Statistical Analyses

The calculation of load–velocity profiles was based on a linear regression approach where the MCV at each load was used to obtain the slope and *y*-axis intercept for each participant. The slope and intercept, and the MVT (for models one and two), were then used as inputs to estimate each participant’s eccentric–concentric and concentric-only bench-press 1RM via five different methods. The first and second methods both used a simple back-calculation of 1RM from the slope, intercept, and MVT data. Specifically, the first model used each participant’s MVT to predict the load they would be able to lift. Similarly, the second model used the slope and intercept along with the group average MVT (~0.17 m/s) to predict the load that an individual would be able to lift. The first calculation method would therefore be a “subject-specific” model, whereas the calculation method would be a “generic” or group model. The third method used multilinear (i.e., ordinary least squares—OLS) regression to predict 1RM load with only the slope and intercept data. The prediction equation for the OLS model is based on minimizing the sum of squares between the actual and predicted 1RM (i.e., the prediction residuals). The fourth and fifth methods used a LASSO regression and an ANN model, respectively, to calculate 1RM from only the slope and intercept data. Thus, the OLS, LASSO, and ANN models did not include MVT as input, which means that the predictions of these models are independent of knowing anything about the minimum velocity with which a person performs a 1RM bench press, either in an eccentric–concentric or concentric-only manner. The LASSO model was cross validated 10 times across 100 ‘lambda’ values, and the model with the lowest error was used for analysis. The regularized regression coefficients for that model were then used to predict 1RM. The ANN consisted of a three-layer feedforward network with a 2-10-1 architecture (two inputs [slope, intercept], 10 hidden neurons, one output (1RM) and used and a sigmoid transfer function between the input and hidden layer and a linear transfer function between the hidden and output layers. The data were randomly split into a training (70%), validation (15%), and test (15%) set. The Levenberg–Marquardt back-propagation algorithm was then used to train the weights and biases of the ANN. The eccentric–concentric and concentric-only 1RM estimates for each participant from each of the five methods were then compiled and used for statistical analysis.

Descriptive data are presented as means ± standard deviations. The estimated 1RM data from each of the five methods were compared against the actual 1RM with paired-samples *t*-tests and Cohen’s *d* effect sizes. Cohen’s *d* effect sizes were interpreted as trivial (0–0.19), small (0.2–0.59), moderate (0.6–1.19), or large (>1.2) [21]. In addition, Pearson’s product-moment correlation coefficients (*r*) were calculated to analyze the association between the estimated 1RM data from each of the five methods and the actual 1RM. Correlation coefficients were interpreted as follows: <0.40 = weak to negligible correlation, 0.40–0.69 = moderate correlation, 0.70–0.89 = strong correlation, 0.90–0.99 = very strong correlation [22]. Lastly, the mean average error (MAE) and standard deviation were calculated for the differences between the estimated 1RM data and the actual 1RM in order to provide a practical indication of the differences in relevant units of measurement (i.e., kg). The five prediction models were implemented in MATLAB R2020a (The MathWorks, Natick, MA, USA), while the statistical comparisons were performed with JASP 0.9.2 (University of Amsterdam, Amsterdam, The Netherlands). All statistical procedures were executed for both types of bench-press (i.e., concentric-only and eccentric–concentric).

## 3. Results

### 3.1. Actual 1RM and Estimated 1RM Comparisons

The actual and estimated 1RM data are presented in Table 2. For the eccentric–concentric bench-press, comparisons between the actual 1RM and estimated 1RM data showed that the two linear regression methods overestimated 1RM (individual-based 1RM: difference = 0.76 kg, *p* = 0.020, *d* = 0.17; group-based 1RM: difference = 0.72 kg, *p* = 0.023, *d* = 0.17). For the concentric-only bench-press, comparisons between the actual 1RM and estimated 1RM data showed that only one of the linear regression methods overestimated 1RM (individual-based 1RM: difference = 0.88 kg, *p* = 0.008, *d* = 0.24).

### 3.2. Actual 1RM and Estimated 1RM Correlations

The correlations between the actual and estimated 1RM data are presented in Table 3. For the eccentric–concentric bench-press, all five estimated 1RM exhibited very strong correlations (*r* = 0.970–0.980) with the actual 1RM, and the mean average errors between the actual 1RM and the estimated 1RM from the five methods ranged from 2.8 to 3.4 kg. For the concentric-only bench-press, all five estimated 1RM exhibited very strong correlations (*r* = 0.987–0.988) with the actual 1RM, and the mean average errors between the actual 1RM and the estimated 1RM from the five methods ranged from 2.7 to 3.1 kg.

### 3.3. 1RM Estimation Equations

The equations used to estimate 1RM data are presented in Table 4. The equations for estimating 1RM with either the individual MVT or the ANN methods are not presented. 

## 4. Discussion

The purpose of the current study was to compare the ability of five different statistical models to estimate eccentric–concentric and concentric-only bench-press 1RM from load–velocity profile data. The results showed that OLS and machine learning methods could accurately estimate the 1RM for both types of bench-press with only limited load–velocity data (i.e., without MVT). The results also showed that linear regression with either individual or group average MVT significantly overestimated the actual 1RM for the eccentric–concentric bench-press. MAE for the estimation of bench press 1RM were comparable for all five methods analyzed, although the OLS and machine learning models produced the smallest errors. These results therefore supported our hypothesis, in that OLS or machine learning methods could be used to effectively estimate athlete’s 1RM from the slope and predicted y-intercept derived from submaximal load–velocity profile data, and without knowledge of the MVT. 

One reported drawback to traditional load–velocity profiling is that to accurately estimate an athlete’s 1RM, the velocity at which the athlete lifts the 1RM load needs to be entered into the linear or polynomial regression equation. To facilitate this process, and not have to perform actual 1RM testing, practitioners sometimes use group-based averages of the MVT, which can be found in the published research literature [6]. However, using an individual’s actual MVT from maximal tests generally provides more reliable results [10,23] because the velocity achieved at different percentages of 1RM, including the velocity at the 1RM itself, is both participant- and exercise-dependent [6,12,24,25]. Moreover, it has been observed that MVT can exhibit poor reliability for different exercises like the back squat, bench-press, or deadlift [8,9,14]. The current study adds to the existing body of the literature about load–velocity profiling by showing that 1RM (MAE ranging 2.7–3.1 kg) can be accurately estimated with the slope and intercept from traditional load–velocity profiles and without MVT. In fact, the errors between the actual and estimated 1RM bench-press in the present investigation were similar to those observed in previous studies on the bench-press exercise [7,19]. 

Interestingly, the 1RM estimations from the linear regression model that used either individual or group average MVT significantly overestimated the actual 1RM for the eccentric–concentric bench-press. In contrast, the 1RM estimations from the OLS or machine learning models, which did not include MVT as input, did not differ significantly from the actual 1RM. These findings suggest that, in the case of the current study, MVT is not necessary to estimate 1RM and that including either individual or group average MVT as an input likely leads to an overfitted model. This idea is supported by the fact that the OLS and LASSO models both had the lowest estimation errors and highest correlation coefficients. Further, the similarity in results from the OLS and LASSO regressions suggests that using only the slope and the intercept from the load–velocity data to estimate bench-press 1RM produces comparably sparse and parsimonious models with better accuracy and generalizability. 

It should be noted that this investigation has some limitations. First, the execution of the bench-press, both in the eccentric–concentric and concentric-only manner, was performed in a Smith machine. Thus, if similar statistical approaches are to be used to estimate the 1RM for other exercises, the variation (i.e., machine-based vs. free weight) should be considered. Second, other intrinsic factors like gender, age, or training status of an individual can affect their load–velocity profile [10,13,15]. For example, males and females exhibit significantly different velocities at each percentage of 1RM, even if they have similar levels of resistance training experience [15]. Additionally, the strength levels of the participants in the present investigation were quite low, with 1RM being equivalent to body mass on average. Future investigations with different exercises and populations may be required to determine if the results of the present investigation are generalizable.

## 5. Conclusions

The present investigation demonstrated that bench-press 1RM can be accurately estimated from load–velocity test data derived from submaximal loads (40–80% 1RM) and without the need to use MVT. In addition, results showed that a relatively simple OLS model can be used to estimate eccentric–concentric and concentric-only bench-press 1RM, and that machine learning models are not necessary for this purpose. Collectively, these results are helpful for strength and conditioning coaches as it supports the practice of estimating 1RM without MVT data.

## Figures and Tables

**Table 1:** Descriptive data of the participants from the two studies from which the load–velocity profiles were obtained.

Study	Sample Size	Age (Year)	Body Mass (kg)	Height (m)
Pestaña-Melero et al., 2018	30	21.2 ± 3.8	72.3 ± 7.3	1.78 ± 0.07
García-Ramos et al., 2020	86	20.9 ± 4.2	74.3 ± 15.6	1.73 ± 0.05

**Table 2:** Actual one-repetition maximum data (1RM) and estimate 1RM data for the eccentric–concentric (Ecc-Conc) and concentric-only (Conc-only) bench-press with five different methods (Ind—individual MVT based estimation, Grp—group average MVT based estimation, OLS—ordinary least squares regression, LASSO—least absolute shrinkage and selection operator regression, ANN—artificial neural network).

Type	Actual 1RM	Estimated 1RM				
		Ind MVT	Grp MVT	OLS	LASSO	ANN
Ecc-Conc	73.7 ± 18.2	74.4 ± 17.2 *	74.4 ± 17.1 *	73.7 ± 17.9	73.7 ± 17.8	73.1 ± 17.0
Conc-only	70.3 ± 22.9	71.2 ± 22.8 *	70.9 ± 21.7	70.3 ± 22.7	70.3 ± 22.7	70.9 ± 21.7

* *p* < 0.05 vs. Actual 1RM; MVT—minimum velocity threshold (i.e., velocity at 1RM).

**Table 3:** Correlations (Pearson’s *r*) and mean average errors (MAE: mean ± SD) between actual one-repetition maximum data (1RM) and estimated 1RM data for the eccentric–concentric (Ecc-Conc) and concentric-only (Conc-only) bench-press with five different methods (Ind—individual MVT based estimation, Grp—group average MVT based estimation, OLS—ordinary least squares regression, LASSO—least absolute shrinkage and selection operator regression, ANN—artificial neural network).

Type		Estimated 1RM				
		Ind MVT	Grp MVT	OLS	LASSO	ANN
Ecc-Conc	*r*	0.970	0.971	0.979	0.979	0.980
	MAE	3.4 ± 4.4	3.4 ± 4.4	2.8 ± 3.7	2.8 ± 3.7	2.9 ± 3.8
Conc-only	*r*	0.987	0.988	0.988	0.988	0.987
	MAE	2.9 ± 3.6	3.1 ± 3.7	2.7 ± 3.5	2.7 ± 3.5	3.1 ± 3.8

MVT—minimum velocity threshold (i.e., velocity at 1RM).

**Table 4:** Equations used to estimate one-repetition maximum data (1RM) and estimated 1RM data for the eccentric–concentric (Ecc-Conc) and concentric-only (Conc-only) bench-press (Grp—group average MVT based equation, OLS—ordinary least squares regression equation, LASSO—least absolute shrinkage and selection operator regression equation).

**Ecc-Conc**	
Grp	1RM = L-V_slope_ × MVT * + L-V_intercept_
OLS	1RM = L-V_slope_ × 0.543 + L-V_intercept_ × 1.250 − 3.721
LASSO	1RM = L-V_slope_ × 0.542 + L-V_intercept_ × 1.249 − 3.711
**Conc-only**	
Grp	1RM = L-V_slope_ × MVT * + L-V_intercept_
OLS	1RM = L-V_slope_ × 0.302 + L-V_intercept_ × 1.128 − 3.749
LASSO	1RM = L-V_slope_ × 0.299 + L-V_intercept_ × 1.125 − 3.735

L-V_slope_—slope of the load–velocity profile; L-V_intercept_—intercept of the load–velocity profile; MVT—minimum velocity threshold (i.e., velocity at 1RM); * Ecc-Conc: MVT = 0.17 m/s; Conc-only: MVT = 1.66 m/s.

## Data Availability

Restrictions apply to the availability of these data. Data was obtained from the original author with the permission.
